# Role of relaxin-2 in human primary osteosarcoma

**DOI:** 10.1186/1475-2867-13-59

**Published:** 2013-06-10

**Authors:** Jinfeng Ma, Min Niu, Wenjiu Yang, Lina Zang, Yongming Xi

**Affiliations:** 1 Department of Spine, the Affiliated Hospital of Qingdao Medical College, Qingdao University, Qingdao 266003, R.P China; 2 Department of pharmacy, the Affiliated Hospital of Qingdao Medical College, Qingdao University, Qingdao 266003, R.P China; 3 Department of Internal medicine-cardiovascular, the Affiliated Hospital of Qingdao Medical College, Qingdao University, Qingdao 266003, R.P China

**Keywords:** Osteosarcoma, Prognosis, Metastases, Angiogenesis, Relaxin-2, Gene treatment

## Abstract

**Background:**

The aim of this study was to clarify the clinicopathological outcome of serum relaxin-2 and tissues relaxin-2 expression levels in human primary osteosarcoma (OS), and to explore the roles of relaxin-2 inhibition and determine its possibility as a therapeutic target in human osteosarcoma.

**Methods:**

Real-time quantitative RT-PCR assay was performed to detect the expression of relaxin-2 mRNA in 36 cases of human osteosarcoma tissue samples. Serum relaxin-2 levels was measured in ELISA-based method in the 36 cases of osteosarcoma and 50 cases of controls. MTT and TUNEL assay was used to detect cell proliferation and apoptosis after relaxin-2 knockdown with siRNA transfection for 48 hs in vitro. Matrigel invasion and angiogenesis formation assay was used to detect cell metastasis and angiogenesis with HMEC-1 endothelial cells after relaxin-2 knockdown with siRNA transfection for 48 hs in vitro. The effects of relaxin-2 knockdown with anti- relaxin-2 mAb treatment on growth, apoptosis angiogenesis formation and lung metastasis in vivo was analyzed.

**Results:**

The results showed the levels of relaxin-2 mRNA expression in osteosarcoma tissue samples were significantly higher than those in the corresponding non-tumor tissue samples (*P* < 0.01), and the serum relaxin-2 levels were significantly higher in OS patients than in healthy controls (*P* < 0.01). The incidence of advanced stage cancer and hematogenous metastasis cancer in the high relaxin-2 mRNA expression group and high serum relaxin-2 levels groups was significantly higher than that in the low relaxin-2 expression group and low serum relaxin-2 levels groups, respectively. Knockdown of relaxin-2 by siRNA transfection in vitro inhibited proliferation, invasion and angiogenesis in vitro in MG-63 OS cells. In vivo, knockdown of relaxin-2 with anti- relaxin-2 mAb treatment inhibited tumor growth by 62% (*P* < 0.01) and the formation of lung metastases was inhibited by 72.4% (*P* < 0.01). Microvascular density was reduced more than 60% due to anti- relaxin-2 mAb treatment (*P* < 0.01).

**Conclusions:**

Our study suggests that overexpression of relaxin-2 is critical for the metastasis of human osteosarcoma. Detection of relaxin-2 mRNA expression or serum relaxin-2 levels may provide the first biological prognostic marker for OS. Furthermore, relaxin-2 is the potential molecular target for osteosarcoma therapy.

## Introduction

One of the most common primary bone tumors in children and adolescents is osteosarcoma, which is most often localized in the metaphysis of the adolescent long bones [1]. Similar to other types of solid tumors, osteosarcoma is characterized by a high propensity for metastasis, especially in lung, which is the main cause of death [2]. Standard treatment for osteosarcoma is surgery and neoadjuvant chemotherapy [3]. Chemotherapy has significantly improved the survival rate from 11% with surgery alone to 60–70% when surgery is combined with chemotherapy [3,4]. Patients with advanced osteosarcoma after front-line chemotherapy usually receive further treatment with additional chemotherapy, which may be considered toxic [5]. Unfortunately, not much progress has been made on improving survival over the past 20 years with regard to the treatment of osteosarcoma. Thus, identification of new targeted therapies is a crucial step forward in the drive towards personalized medicine [3,5,6].

Relaxin is a short circulating peptide hormone. Two highly homologous genes on human chromosome 9 encode relaxin −1 and relaxin −2 peptides with predicted 82% identity at amino acid level [7,8]. Despite having two peptide-coding genes, relaxin gene 1 and 2, the major stored and circulatory form of relaxin in humans is relaxin-2. relaxin-2 is produced in the prostate by males [9] and corpus lutea in females [10], and relaxin-1 is a pseudogene, which does not translate into a functional peptide in rodents, humans and other non-human species.

The effect of relaxin, particularly during pregnancy, is well established in rodents. Levels of relaxin-2 change with the different stages of pregnancy and these patterns are dissimilar across species [11]. Furthermore, relaxin-2 has been shown to increase oocytes fertility [12]. Relaxin-2 has also been shown to be involved in nonreproductive functions [13]. Most recently relaxin-2 has been associated with cancer biology. A number of putative roles, including the modulation of tumor growth, neovascularization, metastasis and oncogenic progression, have been correlated to relaxin overexpression [9,14-16].

Elevated relaxin-2 serum concentrations were found in breast cancer patients, especially in patients with metastatic disease [17]. Stimulation with relaxin-2 increases the invasiveness and migration of breast, endometrial, and thyroid adenocarcinoma cells in vitro accompanied by the up-regulation of matrix metalloproteinase activity and vascular endothelial growth factor expression, which are directly related to cancer progression [18-20].

It has recently found relaxin is able alone to induce the differentiation of PBMCs into mature osteoclasts, suggesting relaxin has an effect on bone metabolism, facilitating the differentiation of osteoclasts [21,22]. Further study showed that relaxin is a potent stimulator of osteoclastogenesis from hematopoietic precursors and regulates the activity of mature osteoclasts, which opened new perspectives on the role of this hormone in bone physiology, diseases and metastasis [22].

The aim of this study was to investigate the relaxin −2 expression in 36 cases of osteosarcoma tissues, and analyzed the association between clinical parameters of osteosarcoma and relaxin −2 expression. Moreover, we address the fundamental question of whether relaxin −2 down-regulation is involved in reducing invasion and metastasis in osteosarcoma cells and inhibition of tumorigenesis.

In the present study, we found that relaxin −2 mRNA was overexpressed in osteosarcoma tissues, and high serum relaxin −2 levels was in osteosarcoma patients. Relaxin-2 mRNA and serum relaxin-2 were significantly higher in advanced stage cancer and hematogenous metastasis cancer. Exogenous down regulation of relaxin-2 suppressed proliferation angiogenesis and migration in MG-63 cells and decreased tumor formation, metastasis and angiogenesis in nude mice. Our study suggests that aberrant expression of relaxin-2 is critical for the development of human osteosarcoma, and relaxin-2 is the better target gene for treatment of osteosarcoma.

## Methods

### Patients and tissue samples

The study was approved by the Research Ethics Committee of Ministry of Public Health of China. Informed consent was obtained from all of the patients. All specimens were handled and made anonymous according to the ethical and legal standards. A total of 36 primary osteosarcoma and corresponding non-tumor tissue samples from the same specimens were collected from the Department of Orthopaedics, the Affiliated Hospital of Medical college, Qingdao university between Jan. 12 - 2006 and Oct. 26 - 2011. None of the patients had received chemotherapy or radiotherapy before surgery. Closed biopsies of all patients were performed by fine-needle aspiration or trephine for diagnosis or/and then surgical treatment. The biopsy samples with bone tissue were decalcified. The pathological diagnosis was performed by the same group of two senior pathologists experienced in osteosarcoma diagnosis. Tumor size data were available based on a review of the imaging studies and the pathology reports. Tumor volume was calculated on the basis of an ellipsoid formula, using the measurements: height × width × depth × 0.52. Patient characteristics were detailed in Table 1. Samples were snap-frozen in liquid nitrogen and stored at −80°C after they were resected.

**Table 1:** **Association between relaxin**-**2 mRNA expression and clinicopathologic factors**

**Clinicopathologic factor**	**Total**	**Serum relaxin-2**		**Relaxin-2 mRNA expression**		
		**(mean ± SEM) (ng/mL)**	**P**-**Value**	**Low(n)**	**High(n)**	**P**-**Value**
**Sex**			0.456			0.568
Female	20	88.24 ± 13.27		7	13	
Male	16	91.58 ± 12.36		8	8	
**Age(Years)**			0.087			0.092
<20	21	95.23 ± 14.16		6	15	
≥20	15	83.62 ± 12.61		9	6	
**Tumor location**			0.562			0.67
Extremity	14	91.54 ± 15.13		6	8	
Axial	22	89.67 ± 14.86		9	13	
**Histologic subtypes**			0.893			0.746
Osteoblastic	12	91.65 ± 13.02		5	7	
Chondroblastic	10	90.54 ± 13.48		4	6	
Fibroblastic	9	89.04 ± 14.23		3	6	
Telangiectactic	5	91.86 ± 13.35		3	2	
**Clinical stage**			0.004			0.018
I + II	20	76. 4 ± 15.27		11	9	
III	16	114.53 ± 17.26		4	12	
**Hematogenous metastasis**			0.001			0.002
No	15	70.53 ± 10.42		11	4	
Yes	21	108.6 ± 14.87		4	17	
**Grade**						0.742
High	30	89.12 ± 13.90	0.642	11	19	
Moderate	3	94.40 ± 12.16		2	1	
Low	3	96.18 ± 12.45		2	1	

### ELISA-based measurement of serum relaxin-2

Concentrations of relaxin-2 in serum samples of 36 patients and 50 healthy individuals with equivalent distribution of age and sex were determined using relaxin-2-specific enzyme-linked immunosorbent assay kit (ELISA, yfswbio, Shanghai, China) according to the manufacturer’s protocol. Briefly, the sera were added in duplicate to the wells of the microtiter plate coated with an antibody against relaxin-2 with horseradish peroxidase-conjugate. Then, absorbance at the wave length of 450 nm in each microwell was measured using a Victor3 1420 Multilabel Counter reader (Perkin-Elmer LAS GmbH, Rodgau, Germany). Amounts of relaxin-2 were calculated using standards included in the ELISA kit and software supplied with Victor3. The lower limit of quantification was 0.1 ug/l serum sample. The inter-and intraassay coefficients of variation was 15 and 10.5% respectively.

### RNA preparation and quantitative RT-PCR assay

Total RNA was extracted using Trizol (GIBCO/BRL), and mRNA was isolated from total RNA by the FastTrack mRNA isolation kit (Invitrogen). RNAs were quantified by UV absorption measurements and stored at −80°C. Isolated RNA was reverse transcribed into cDNA using the Takara RNA PCR Kit (AMV) Ver.2 (Takara, Ohtsu, Japan) according to manufacturer’s instructions. Real-time quantitative PCR was done using the ABI Prism 7700 Sequence Detection System (Perkin-Elmer Applied Biosystems). Briefly, each PCR mixture contained 1 μl of cDNA, TaqMan Universal PCR master mix (Perkin-Elmer Applied Biosystems), primer pair, and TaqMan probe in a final volume of 50 μl. The PCR conditions were an initial denaturation step of 2 min at 50°C and 10 min at 95°C, followed by 33 cycles consisting of 15 s at 95°C, and a 1 min at 60°C. Quantitative normalization of Relaxin-2 cDNA in each sample was done using β-actin as an internal control. Real-time PCR assays were done in triplicate, and the mean values were used for calculations of mRNA expression. Equal amounts of cDNA from each sample were amplified using the following primers to detect the expression of Relaxin-2: sense: 5′-TGCTGCGCTGGTTCTTGTCC′-3′,reverse: 5′-ACCGTGTTGCTATCCTCAGTTCCT-3′ with an expected size of 306 base pairs (bp). Quantitative real-time RT-PCR assay was performed to detect β-actin expression that was used to normalize the amount of cDNA for each sample. β-actin primers were as follows: sense: 5′-GTGCGTGACATTAAGGA-3′, reverse: 5′-CTAAGTCATAGTCCGCC-3′ (520 bp). Cut-off point selection for the Relaxin-2 mRNA was performed by searching for a cut point yielding the smallest log-rank P-value and divided to the higher and lower Relaxin-2 mRNA expression levels.

### Cell lines and reagents

The human osteosarcoma cell lines MG-63, U-2OS and Saos-2 were obtained from the ATCC (Rockville, MD), and incubated in RPMI 1640 medium containing 10% fetal calf serum (FCS, Gibco) and 1% antibiotics (P/S, penicillin 10.000 U/ml and streptomycin 10.000 mg/ml, in 75 cm^2^ culture flasks (Falcon, Mountain View, CA) until they had formed a confluent monolayer. Relaxin 2 siRNA (h) was acquired from Santa Cruz. MAb to Relaxin 2 was from Biodesign (Shanghai, China).

### Transfection with small interfering RNA (siRNA)

Relaxin-2 siRNA was acquired from Santa Cruz, Shanghai, China. Cells (1 × 10^5^ cells) MG-63 cells were maintained in Dulbecco's modified Eagle's medium (DMEM) supplemented with 10% fetal bovine serum, penicillin (100 U/ml) and streptomycin (100 mg/ml). One day before transfection, cells were trypsinized and resuspended in DMEM without antibiotics, and plated into a 24-well plate at a density of 1.5 × 10^5^ per 0.6 ml per well. Relaxin-2 siRNA was performed using Lipofectamine RNAiMAX (Invitrogen) according to the manufacturer's protocol. A control green-fuorescent protein (GFP) siRNA was as a control. A 10 nM concentration of siRNA was used for the transfections. 72-h after transfection, the cells were harvested for western blot analysis of the knockdown level of the exogenous proteins by siRNA.

### Western blot analysis

Cells were harvested, pelleted by centrifugation, washed with ice-cold PBS, and lysed with RIPA buffer (150 mM NaCl, 50 mM Tris base pH 8.0, 1 mM EDTA, 0.5% sodium deoxycholate, 1% NP-40, 0.1% sodium dodecyl sulfate, 1 mM DTT, 1 mM PMSF, and 1 mM Na3VO4) supplemented with protease inhibitor. Protein concentration of the supernatants of cell extract was determined using a BCA protein assay kit (Pierce Biotechnology, Inc.). Equal amounts of proteins were loaded on 10% SDS-polyacrylamide gel. After electrophoresis, the proteins were transferred to PVDF membranes, and the blots were subsequently probed with the following antibodies: Relaxin-2 (1:100) and anti-β-actin (C-2, used as a total protein loading control)were all purchased from Santa Cruz Biotechnology, Santa Cruz, CA, USA). For detection, horseradish peroxidase-conjugated secondary antibodies were used (1:500) followed by enhanced chemiluminescence development(Millipore). Normalization of the results was done by running parallel Western blots using actin as control. The optical density was quantified using an AlphaEase FC Version4 analysis software (Alphalmager HP, Alpha Innotech, San Leandro, CA, USA).

### Cell proliferation assay

The effect of Relaxin 2 sliencing on proliferation was demonstrated using MTT assay. In brief, cells transfected with relaxin 2 siRNA for 24 hours were seeded into 96-well plates at 1 × 10^4^ per well. After incubation for 24, 48, 72, or 96 h, 200 uL of MTT reagent (5 mg/mL; Sigma) was added to each well. Plates were incubated at 37°C for 4 h and then the supernatant was removed and 200 uL of dimethyl sulfoxide (Sigma) was used to dissolve the resultant formazan crystals. The absorption was read at 490 nm using a spectrophotometer. The MTT assay was performed in triplicate.

### Tunel staining

Cells transfected with relaxin 2 siRNA for 48 hs were plated on polylysine-coated slides, fixed with 4% paraformaldehyde in 0.1 M phosphate-buffered saline (PBS) for 1 h at 25°C, rinsed with 0.1 M PBS, pH 7.4, and permeabilized with 1% Triton X-100 in 0.01 M citrate buffer, pH 6.0. DNA fragmentation was detected using an ApopTag Kit (Chemicon international, Temecula, CA) according to the manufacturer’s instructions. The percentage of apoptotic cells was calculated as the number of apoptotic cells per number of total cells × 100%.

### Matrigel invasion assay

The invasiveness of OS cells was tested after transfection (48 hs) as previously described. The MG-63 cells (1 × 10^6^/mL) were added to the upper wells coated with Matrigel with serum-free medium containing 25 ug/mL fibronectin as a chemoattractive agent in the lower wells. After a 24-h incubation period, cells that migrated through the filters into the lower chamber were counted by the number of cells on the lower side of the membrane in five random fields after staining with Hema-3 kit.

### In vitro angiogenesis assay

MG-63 cells (2 × 10^4^/mL) were transfected with relaxin 2 siRNA for 48 h and the conditioned medium was filtered off for future research. HMEC-1 endothelial cells (4 × 10^4^) were seeded onto eight-well chamber slides and the aforementioned conditioned medium was added. Cells were cultured for 72 h until capillary network formation was observed. The number of branch points and total number of branches per point were counted after H&E staining to quantify the degree of angiogenesis

### Animal studies

Male SCID mice, each 8 weeks old and weighing 20–25 g, were obtained from Qingdao Medical college, Qingdao University for tumor implantation. All animals were maintained in a sterile environment and cared for within the laboratory animal regulations of the Ministry of Science and Technology of the People’s Republic of China (http://www.most.gov.cn/kytj/kytjzcwj/200411). Full details of the study approval by the ethics committee at the affiliated hospital of medical college, Qingdao University. MG-63 cells were trypsinized, washed with serumfree MEM, and resuspended in PBS, after which their concentration was adjusted to 2 × 10^4^ cells/100 uL in PBS. To generate orthotopic xenograft models, these cell suspensions were injected into the anterior tibialis muscle of the SCID mice. After 24 h, these mice received intraperitoneal injections of 100 uL of PBS containing 100 ug of anti- relaxin-2 mAb (Cellmid Limited) (n = 5) or PBS alone (n = 5); the mice received this same treatment every 5 days for 42 days. Tumor development in individual animals (n = 5/group) was assessed every 7 days by means of sequential measurements of the bilateral difference in calf circumferences. Mice were killed 42 days after injections of cells, and the tumors were harvested. For the model of settlement of tumor cells to lung, cells transfected with Relaxin-2 siRNA were trypsinized, washed, and resuspended in PBS, and their concentration was adjusted to a concentration of 1 × 10^5^ cells/100 uL in PBS. MG-63 cell suspensions were then injected into the tail vein of SCID mice, followed by analysis of settlement of tumor cells to lung after 2 weeks. Micro-metastasis were counted through the whole lung section on slides using a microscope and quantified.

### Microvessel density

The tumor vasculature was stained with an antibody against CD31, and microvessel density (MVD) was determined by counting CD31-stained vessels of tumor slides by examining “hotspots” according to the manufacture’s instruction. Vessel density per 200 field was quantified from 6 to 8 fields per tumor section from the treatment and control groups and expressed as percentage per area (200× field). Percentage per area was assessed using ProImage software.

### Statistical analysis

All experiments were conducted in triplicate and carried out on three or more separate occasions. Data presented are means of the three or more independent experiments ± SE. Statistically significant differences were determined by *X*^2^ and Student’s t test and were defined as ^*^P < 0.05. All analyses were performed with SPSS version 13.0 software.

## Results

### Overexpression of relaxin-2 mRNA in osteosarcoma tissue samples

**Figure 1:**
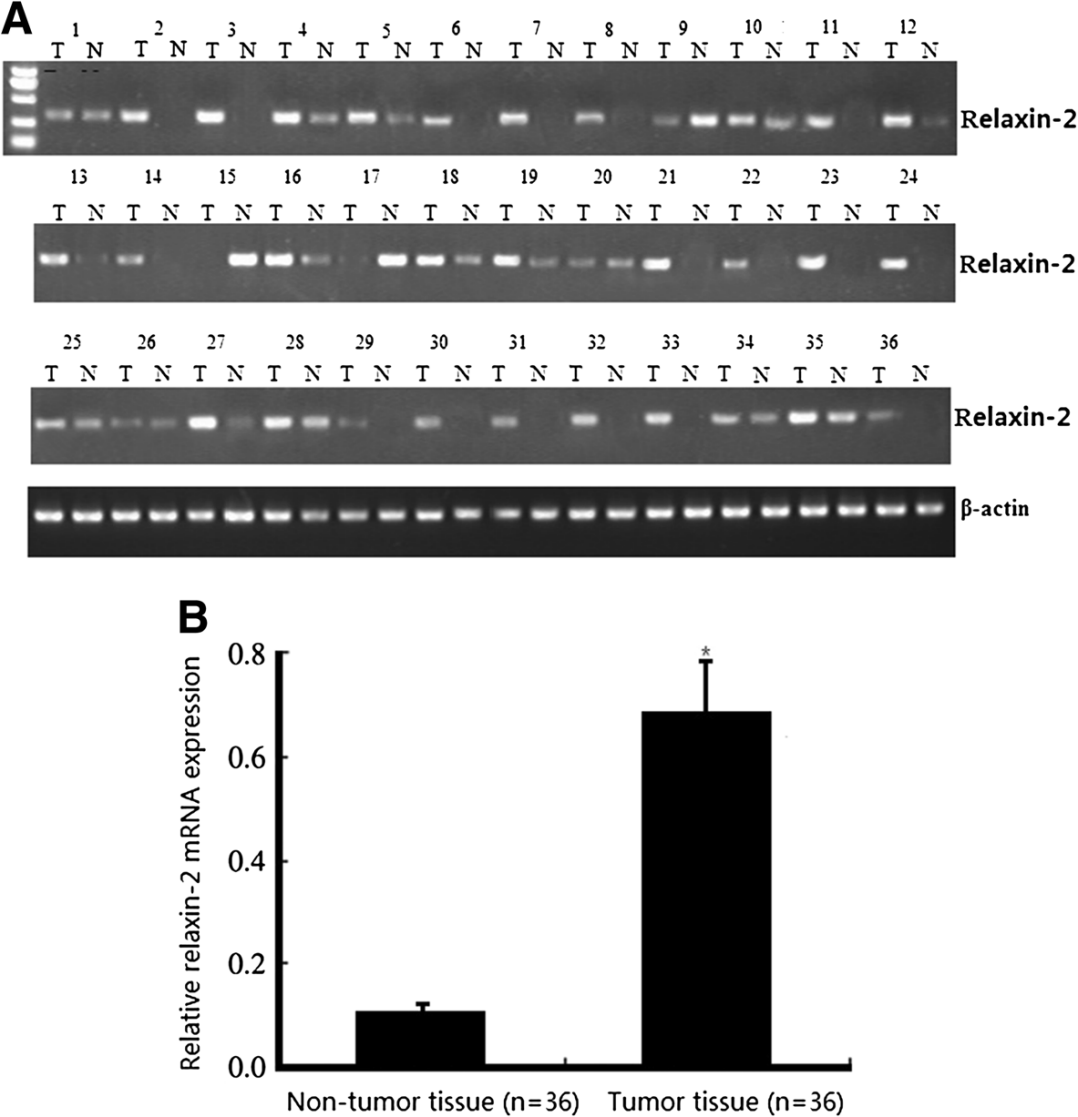
**Detection of relaxin-2 mRNA expression in tissue samples. A**. Gel images of electrophoresis. **B**. Real-time quantitative RT-PCR assay. Osteosarcoma tissues showed obviously higher relaxin-2 mRNA expression than corresponding non-tumor tissues. β-actin was used to normalize for any differences in mRNA between lanes. Student’s t-test showed a significant difference (*P < 0.01).

Real-time quantitative RT-PCR assay was performed to detect the expression of relaxin-2 mRNA in osteosarcoma tissues or corresponding non-tumor tissues from 36 osteosarcoma patients. As shown in Figure 1A, the levels of relaxin-2 mRNA expression in osteosarcoma tissue samples were significantly higher than those in the corresponding non-tumor tissue samples, which showed no or very low levels of relaxin-2 mRNA expression. Moreover, the average level of relaxin-2 mRNA (0.743 ± 0.12) in tumor tissues was significantly higher than that in corresponding non-tumor tissues (0.106 ± 0.06; Figure 1B, P < 0.01). Additionally, patients with relaxin-2 mRNA expression levels in tumor tissues less than 0.392 (average value) were considered as the low expression group (n = 15), and patients with relaxin-2 mRNA expression levels in tumor tissues equal to or greater than 0.392 were considered as the high expression group (n = 21).

### Serum levels of relaxin-2

ELISA assay was performed to detect the serum relaxin-2 levels in 36 osteosarcoma patients and 50 controls. In healthy donors, serum levels of relaxin-2 were 11.47 ± 3.24 ng/ml (mean ± SEM) ranging from 0.28 to 16.74. ng/ml. In OS patients, the corresponding values were 90.37 ± 13.56 ng/ml (mean ± SEM) ranging from 8.6 to 132.5 ng/ml. The serum relaxin-2 levels were significantly higher in OS patients than in healthy controls (*P* < 0.01).

### The correlation between relaxin-2 mRNA or serum relaxin-2 levels and clinical pathological stages

We evaluated the correlation between relaxin-2 mRNA expressions in the OS tissues with clinical pathological stages as shown in Table 1. The incidence of advanced stage cancer in the high relaxin-2 mRNA expression group (12 of 16, 75%) was significantly higher (P = 0.018) than that in the low relaxin-2 expression group (9 of 20, 45%), and the incidence of hematogenous metastasis in the high relaxin-2 mRNA expression group (17 of 21, 81%) was significantly higher (P = 0.002) than that in the low relaxin-2 expression group (4 of 25, 16%). However, there were no associations between relaxin-2 mRNA expression and other factors including gender, age, grade, tumor location or histology (*P* = 0.568, 0.092, 0.549, 0.742 and 0.746, respectively). Similar to relaxin-2 mRNA, serum relaxin-2 were significantly higher in advanced stage cancer and hematogenous metastasis cancer (Table 1). There were no associations between relaxin-2 levels and other factors including gender, age, grade, tumor location or histology, respectively.

### Expression of relaxin-2 in human osteosarcoma cell lines

**Figure 2:**
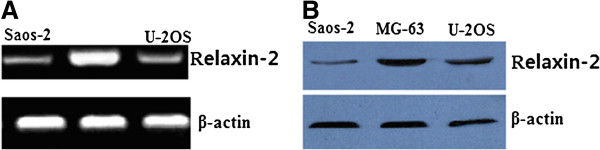
**Detection of relaxin-2 mRNA and proterin expression in human osteosarcoma cell lines. A**. Gel images of electrophoresis. **B**. Western blot assay. MG-63 cell line had the highest expression level of relaxin-2 mRNA and protein. Osteosarcoma tissues showed obviously higher relaxin-2 mRNA expression than corresponding non-tumor tissues. β-actin was used to normalize for any differences in mRNA between lanes.

Relaxin-2 protein and mRNA was examined in 3 human osteosarcoma cell lines MG-63, U-2OS and Saos-2 by western-blot and QRT-PCR. The results showed that the MG-63 cell line had the highest expression level of relaxin-2 mRNA (Figure 2A) and protein (Figure 2B). U-2OS and Saos-2 cells displayed low expression levels. In this regard, the cell line MG-63 was chosen for further studies.

### Down-regulation of relaxin-2 in human osteosarcoma MG-63 cell line

**Figure 3:**
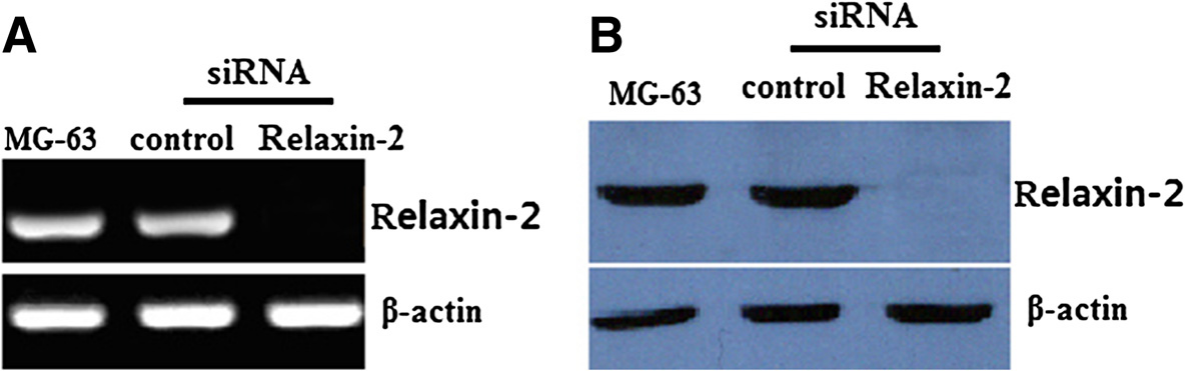
**Effects of relaxin-2 mRNA on relaxin-2 mRNA and protein assessed by quantitative RT-PCR and western blot assays.** Relaxin-2 mRNA (**A**) and protein (**B**) was completely inhibited by relaxin-2 siRNA transfection for 48 hs than those of MG-63-non-transfection or MG-63-siRNA control cells respectively.

The cell line MG-63 was transiently transfected with either relaxin-2 siRNA or the control siRNA for 48 h, then the cells were lysed and processed for mRNA and protein analysis. Relaxin-2 siRNA transfectants showed a remarkably reduced expression of relaxin-2 mRNA (Figure 3A) and protein (Figure 3B) levels. These observations provided direct evidence that relaxin-2 siRNA transfection could effectly repressed relaxin-2 expression in human osteosarcoma MG-63 cell lines.

### Effect of relaxin-2 knockdown on osteosarcoma MG-63 cell proliferation and apoptosis

**Figure 4:**
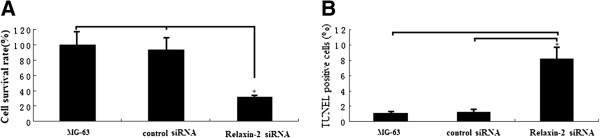
**Effect of relaxin-2 knockdown on osteosarcoma MG**-**63 cell proliferation and apoptosis. A**, Cell viability was determined by MTT. The growth rate of MG-63 cells after relaxin-2 knockdown with siRNA for 48 hs was significantly decreased in MTT assay. **B**, Apoptotic cells was determined by TUNEL analysis in MG-63 cells following relaxin-2 knockdown with siRNA for 48 hs. The apoptotic cells in relaxin-2 siRNA transfectants were significantly higher compared to MG-63-non-transfection or MG-63-siRNA control cells respectively. Columns, mean values from triplicate experiments; Each bar represents mean ± SE (n = 3); ^*^, *P* < 0.05 versus MG-63-non-transfection or MG-63-siRNA control.

We next investigated the role of relaxin-2 in osteosarcoma by means of relaxin-2 knockdown with siRNA. As Figure 4A shows, relaxin-2 knockdown by siRNA for 48 hours inhibited MG-63 cell proliferation significantly by MTT assay compared to MG-63-non-transfection or MG-63-siRNA control cells respectively.

Apoptosis was quantified by TUNEL analysis. Following relaxin-2 knockdown with siRNA for 48 hs, the apoptotic MG-63 cells was 8.4%, more than that of MG-63-non-transfection (1.2%) or MG-63-siRNA control (1.3%) cells respectively, which showed significant difference in their apoptotic response (Figure 4B). The results indicates that transfection might, to a large degree, inhibit proliferation by inducing apoptosis in OS cells.

### Effect of relaxin-2 knockdown on osteosarcoma MG-63 cell invasion

**Figure 5:**
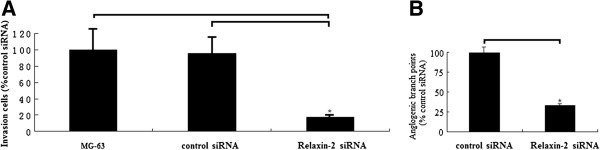
**Effect of relaxin-2 knockdown with siRNA on invasion and angiogenesis. A**, invasiveness of MG-63 cells transfected with relaxin-2 siRNA in Matrigel invasion assay. Cells were transfected with mock siRNA or relaxin-2 siRNA. After 48 h, 1 × 10^5^ cells were allowed to invade through transwell inserts (8 Am) coated with Matrigel. The cells on lower surface of chambers were stained, counted, and photographed under a light microscope. The in vitro inhibition of invasiveness was calculated in five random 200-fold fields using the following formula: percent inhibition = (mock siRNA-relaxin-2 siRNA) /mock. Columns, mean from three separate experiments; bars, (^*^P < 0.01). **B**, HMEC endothelial cells treated with conditional medium from MG-63 cells after siRNA transfection by in vitro angiogenesis assay. The conditioned medium of MG-63 cells was collected after 48-h transfection following filtering of medium. HMEC-1endothelial cells seeded in eight-chamber slides were cultured with the above medium for 72 h until the formation of capillary network was observed. In the end of the experiment, angiogenesis was assessed by H&E staining and photographed under a microscope. columns, mean from three separate experiments; bars, SD (^*^P < 0.01).

The result (Figure 5A) from Matrigel invasion assay indicates that relaxin-2 knockdown with siRNA significantly inhibited the invasion of MG-63 cells by 83%, as compared with mock-transfected and MG-63-non-transfected cells. Furthermore, the results (Figure 5B) showed that HMECs treated with conditioned media from mock transfected MG-63 cells were able to form capillary-like structures. It shows that HMECs treated with conditioned media from relaxin-2 siRNA -transfected MG-63cells showed less capillary-like networks (63%), as compared with the controls.

### Effect of relaxin-2 inhibition with anti- relaxin-2 mAb on tumor growth in nude mice

**Figure 6:**
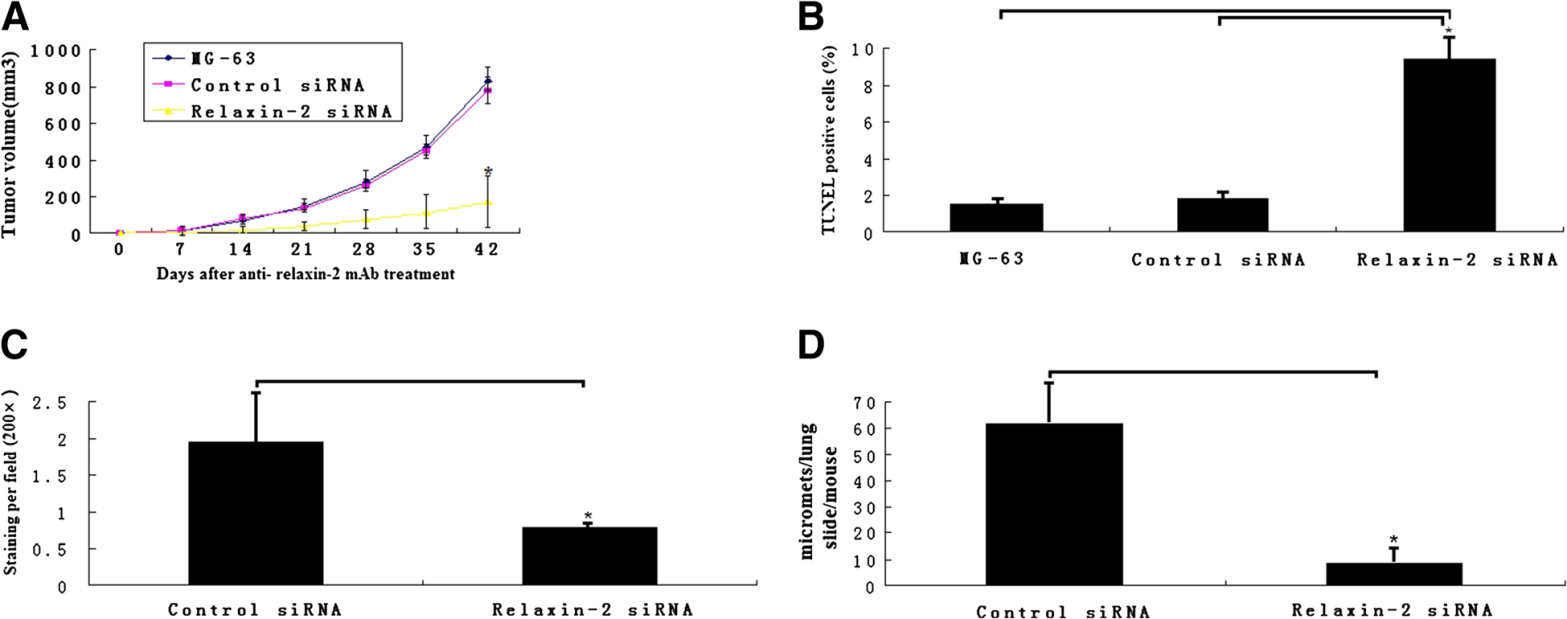
**Tumor growth inhibition and prevention of metastasis and angiogenesis formation by relaxin**-**2 inhibition. A**, treatment with anti- relaxin-2 mAb at 100 ug weekly i.p for 42 d. inhibited primary tumor growth by 73 ± 6% compared with controls (*n* = 6; ^*^P <0.01). **B**, treatment with anti- relaxin-2 mAb at 100 ug weekly i.p for 42 d. promotes apoptosis in primary tumor cells by 8.2 ± 1.2% compared with controls (*n* = 6; ^*^P <0.01). **C**, inhibition of tumor angiogenesis by anti- relaxin-2 mAb treatment as measured by MVD. The vascular density of the anti- relaxin-2 mAb–treated tumor was 0.57 ± 0.22%, which was significantly lower than the controls 1.84 ± 0.26%.( *n* = 6). *, *P* < 0.01. **D**, MG-63 cells transfected with Relaxin-2 siRNA (1 × 10^5^ cells/100 uL in PBS) were injected into the tail vein of SCID mice, followed by analysis of settlement of tumor cells to lung after 2 weeks. The number of macroscopic tumor nodules in Relaxin-2 siRNA–treated mice was 9.46 ± 1.8 lung nodules per mouse versus 62.4 ± 7.4 nodules in the controls (*n* = 5 mice/group). One way ANOVA analysis was performed to compare the number of micro-metastasis in the lung of mice. ^*^, *P* < 0.05.

Having shown that relaxin-2 knockdown with siRNA decreased invasion and angiogenesis in MG-63 cells in vitro, we tested the growth of the tumors accomplished by anti- relaxin-2 mAb on tumor growth in nude mice. As shown in Figure 6A, the tumors MG-63 cells treated with anti- relaxin-2 mAb for 42 d showed a significant reduction of tumor volum as compared with control groups, whereas MG-63 cells developed obvious tumors in nude mice. Analyzing tissue sections from tumors showed that anti- relaxin-2 mAb significantly increased apoptosis in the tumors as assessed by TUNEL staining (Figure 6B). Furthermore, we analyzed the tumor tissues from control and anti- relaxin-2 mAb groups for relaxin using Western blot assay. We observed significant expression levels of relaxin-2 in control tumor sections. However, expression levels were drastically reduced in the tumor sections of mice treated with anti- relaxin-2 mAb (data not shown). These data show that relaxin-2 inhibition effectively inhibits the growth of OS tumors through apoptosis induction.

### Relaxin-2 inhibition with anti- relaxin-2 mAb *decreased MVD*

To determine whether relaxin-2 blockade has an effect on MG-63 tumor vasculature, MVD was analyzed. New blood vessel formation was significantly reduced in MG-63 tumor by anti- relaxin-2 mAb in the intervention model (80% inhibition). The percentage area occupied by vasculature in anti- relaxin-2 mAb–treated tumors was 0.57 ± 0.22% versus 1.84 ± 0.26% in controls (*n* = 8; *P* < 0.01; Figure 6C).

### Relaxin-2 knockdown by siRNA diminished lung metastasis

Mice with MG-63/ relaxin-2 siRNA cells were sacrificed on the same day of tumor injection (14 days), lungs were harvested and lung sections were examined for cancer cell micrometastasis. The number of macroscopic tumor nodules in relaxin-2 siRNA-treated mice was 9.46 ± 1.8 lung nodules per mouse versus 72.4 ± 7.4 nodules in the controls (*n* = 5 mice/group). *, *P* < 0.01 (Figure 6D).

## Discussion

In the present study, we found that the expression of relaxin-2 mRNA were significantly higher in osteosarcoma tissue samples than those in corresponding non-tumor tissue samples. Moreover, the higher expression levels of relaxin-2 mRNA were significantly correlated with clinical stage and the status of hematogenous metastasis but not other clinicopathological factors. Thus, we conclude that relaxin-2 may play important roles in osteosarcoma development and metastasis, which is also consistent with previous reports in other malignancies [9,14-16,18,19].

It has found by Binder et al. [17] relaxin-2 concentrations in cancer patients were significantly higher than in a control population of healthy blood donors and patients with various other diseases. There was a significant difference between patients with metastases and those without, irrespective of their hormonal status. Our results demonstrate that relaxin-2 serum concentrations in a population of osteosarcoma patients yielded significantly higher relaxin-2 concentrations than in a control group of healthy blood donors. relaxin-2 concentrations were elevated especially in patients with high clinical stage and the status of hematogenous metastasis.

We therefore suggested that the expression of relaxin-2 mRNA in osteosarcoma tissues or relaxin-2 serum concentrations in a population of osteosarcoma patients may serve as a new prognostic predictor for osteosarcoma. Relaxin-2 may be the better target gene for treatment of osteosarcoma.

It has recently found adenoviral-mediated delivery of prorelaxin-2 gene increases the invasiveness of canine breast cancer cells [16]. The lentiviral delivery of relaxin-2 into PC-3 prostate cancer cells increases xenograft tumor growth [15]. It was shown that relaxin-2 is a direct downstream target of R273H p53 mutation in prostate carcinoma cells [23], and relaxin expression is up-regulated by androgen withdrawal in vitro and in vivo [24]. Relaxin is also a potent stimulator of osteoclastogenesis from hematopoietic precursors and regulates the activity of mature osteoclasts [22], they did not investigate whether relaxin has a potent effect on osteoblasts.

MG-63 is the osteoblast, it has the osteoblast-like features. Relaxin-2 was overexpressed in the MG-63 cells. In the present study, we investigate whether relaxin-2 has a potent effect on MG-63 cells. To investigate the potential of relaxin as an effective therapeutic target for osteosarcoma gene therapy, we employed RNA interference to knockdown the endogenous relaxin expression in osteosarcoma cells, which showed that relaxin downregulation could inhibit the proliferation capacity of osteosarcoma cells. Moreover, relaxin downregulation could induce apoptosis enhancement in osteosarcoma cells. The results indicates that transfection might, to a large degree, inhibit proliferation by inducing apoptosis in osteosarcoma cells in vitro.

Previous studies have shown that knockdown of relaxin-2 inhibited invasion and angiogenesis in cancer cells in vitro [15,20]. In this study, we show that relaxin-2 suppression by siRNA inhibited MG-63 cell migration by migration assay. Relaxin was also shown to induce vascular endothelial growth factor and procollagenase expression in human endometrial cells and to stimulate blood vessel formation in the primate endometrium [25,26]. We show here that suppression of relaxin-2 gene reduces the endothelial tube formation of HUVEC formation or capillary-like structures in HMECs. Thus, relaxin-2 inhibition may act anti-angiogenic by a direct effect on endothelial cells. These data provide evidence that the relaxin-2 gene may be associated with proliferation, angiogenesis, invasion, and metastatic spread of cancerous cells during progression of human osteosarcoma in vitro.

It has shown above inhibition of relaxin-2 involved in the growth, invasion, metastasis, and angiogenesis of human osteosarcoma cells in vitro. We then studied the effect of inhibition of relaxin-2 on osteosarcoma cells in vivo mice modes. In this report, we demonstrate that relaxin-2 suppression by anti- relaxin-2 mAb can greatly inhibit the expression of relaxin-2 protein and suppress the tumorigenic phenotype of MG-63 cells inoculated s.c. in nude mice. When injected subcutaneously in nude mice, all xenograft tumours derived from anti- relaxin-2 mAb treated MG-63 tumors contained reduced or almost undetectable levels of relaxin-2 protein when compared with the control tumour tissues. Additionally, we showed that the suppression of relaxin led to a significant increase in cancer cell apoptosis.

Additional functions of relaxin-2, such as stimulation of angiogenesis [15], might have also contributed to cancer growth. In the present study, inhibit expression of relaxin-2 by anti- relaxin-2 mAb was observed significant decreases in the expression of CD31. It reduced the MG-63 tumor vasculature, the new blood vessel formation was significantly reduced in MG-63 tumor by anti- relaxin-2 mAb in the intervention model (80% inhibition), which suggested that relaxin-2 blockage inhibits tumor growth by in part inhibition of angiogenesis.

Osteosarcoma is characterized by a high propensity for metastasis, especially in lung, which is the main cause of death [2]. In vitro assays showed that relaxin-2 siRNA treatment was indeed able to reduce significantly tumor invasiveness. To investigate whether the relaxin-2 knockdown can reduce the metastatic burden in MG-63 tumor, MG-63/ relaxin-2 siRNA cells were injected to the mice by tail. Mice with MG-63/ relaxin-2 siRNA cells were sacrificed on the same day of tumor injection (14 days), lungs were harvested and lung sections were examined for cancer cell micrometastasis. The results showed the number of macroscopic tumor nodules in relaxin-2 siRNA-treated mice was significantly decreased, and the effects did reach statistical significance. This result is in contrast with the findings of the in vitro assays, in which relaxin-2 inhibition was indeed able to inhibit invasion

In summary, our data demonstrated that relaxin −2 was overexpressed in osteosarcoma tissues and patients’. Relaxin-2 mRNA and serum relaxin-2 were significantly higher in advanced stage cancer and hematogenous metastasis cancer. Downregulation of relaxin-2 by siRNA or anti- relaxin-2 mAb on MG-63 effectively suppressed tumor growth in vitro or vivo through increased apoptosis and decreased proliferation of MG-63 cancer cells. Furthermore, the suppression of relaxin-2 significantly reduced invasion, lung metastasis, and angiogenesis in vitro or vivo. We therefore suggested that relaxin-2 may be as a potential therapeutic targets for osteosarcoma treatment. However, the mechanisms of relaxin-2 effects in osteosarcoma cells remain to be further elucidated.

## Competing interests

All authors declare that they have no competing interests.

## Authors’ contributions

JM, LZ and WY mainly carried out the Real-time quantitative RT-PCR assay, and drafted the manuscript. MN and YX carried out the other molecular assay. All authors read and approved the final manuscript.
